# Effects of kinect-based virtual reality training on bone mineral density and fracture risk in postmenopausal women with osteopenia: a randomized controlled trial

**DOI:** 10.1038/s41598-024-57358-7

**Published:** 2024-03-20

**Authors:** Saima Riaz, Syed Shakil Ur Rehman, Sana Hafeez, Danish Hassan

**Affiliations:** 1 Department of Physical Therapy, Riphah College of Rehabilitation and Allied Health Sciences, Riphah International University, Gulberg III, Lahore, 54000 Pakistan; 2 Riphah College of Rehabilitation and Allied Health Sciences, Riphah International University, Lahore, 54000 Pakistan; 3 School of Health Sciences, University of Management and Technology, Lahore, 54000 Pakistan

**Keywords:** Health care, Health occupations, Rheumatology

## Abstract

Osteopenia is a condition characterized by low bone mineral density (BMD) that increases fracture risk, particularly among postmenopausal women (PMW). This study aimed to determine the effects of Kinect-based VRT on BMD and fracture risk in PMW with osteopenia. The study was a prospective, two-arm, parallel-design, randomized controlled trial. The study enrolled 52 participants, 26 randomly assigned to each group. In the experimental group, Kinect-based VRT was provided thrice weekly for 24 weeks for 45 min/session. Both groups were instructed to engage in a daily 30-min walk outdoors. The fracture risk assessment tool (FRAX) was used to calculate fracture risk, and dual-energy X-ray absorptiometry was used to measure lumbar spine and femur neck BMD. Both variables were assessed at baseline and 24 weeks afterwards. After 24 weeks of Kinect-based VRT, the experimental group showed significant BMD increases in the right and left femoral necks and lumbar spine (*p* value < 0.001). In the control group, the BMD at the right and left femoral necks showed fewer significant changes (*p* value < 0.022 and 0.004, respectively). In the control group, lumbar spine BMD did not change (*p* = 0.57). The experimental group showed significantly lower FRAX scores for hip fracture prediction (HFP) and hip prediction of major osteoporotic (HPMO) at both femoral necks (*p* value < 0.001) than the control group (*p* = 0.05 and *p* = 0.01, respectively), but no significant change at the left femoral neck for HFP (*p* = 0.66) or HPMO (*p* = 0.26). These findings indicate that a Kinect-based VRT intervention resulted in significantly increased BMD and a reduced fracture risk, as predicted by HFP and HPMO measurements. These improvements were more pronounced in the experimental group than in the control group. Thus, Kinect-based VRT may be utilized as an effective intervention to improve BMD and reduce fracture risk in postmenopausal women with osteopenia.

## Introduction

Bone mineral density (BMD) is a measurement of the mineral content of bones. It is essential in determining bone strength and is used to detect osteopenia and osteoporosis. Osteopenia is a condition in which BMD is lower than normal but not low enough to be diagnosed as osteoporosis. Osteopenia is characterized by a steady decrease in BMD and is associated with an increased risk of osteoporosis and bone fractures^1^. According to the WHO, BMD loss with a T score between − 1 and − 2.5 is a global health issue that reduces quality of life. After 35, bone loss increases gradually, and osteoporosis grows exponentially after menopause^2^.

Osteoporosis affects 7.2 million women and 9.9 million adults in Pakistan. Furthermore, the projected 40 million Pakistanis with osteoporosis were evenly divided between men and women. The number of Pakistanis who have osteoporosis is expected to climb, reaching 11.3 million in 2020 and 12.9 million in 2050^3^. Age, oestrogen levels, calcium and vitamin D intake, smoking, and physical activity can all impact BMD and fracture risk in PMW with osteopenia. Weight-bearing, resistance, and high-impact exercise programs were found to enhance BMD and reduce the fracture risk in this population. Exercise has also been demonstrated to increase muscle strength, balance, and coordination, which are crucial for reducing the risk of fractures and falls^4^.

DXA, the diagnostic gold standard, is costly and challenging in many Asian nations. Asia will account for almost half of osteoporotic hip fractures by 2050. Asia’s high osteoporosis rate is caused by low calcium intake, vitamin D inadequacy, increased longevity, gender inequity, early menopause, hereditary susceptibility, a lack of diagnostic resources, bone health awareness, altered food habits, and lifestyle changes^5^.

The rate of bone loss in PMW increases with age, with annual losses of 0.6%, 1.1%, and 2.1% for the age Groups 60–69, 70–79, and > 80, respectively. More specifically, the loss in the first 4–5 years is 1.5% each year for the spine and 1.1–1.4% for the femoral neck. Because bone loss is more rapid in the early postmenopausal era, it is slower in subsequent years^6^. Osteoporosis treatment must involve drugs to improve BMC and BMD. Depending on the drug and demographics, it reduces osteoporosis by 20–60%^7^. Postmenopausal hormone or pharmacologic therapy can treat osteoporosis over the long term. Much criticism has been made about its safety^8^. Hormonal therapy may affect female well-being because exogenous substances produce complex physiological changes. Hypercoagulability from chemical therapy can cause stroke and venous thromboembolism, according to one study^9^. It has been determined that medications have no direct effect on the primary risk factors for fractures, such as muscle strength, muscle power, endurance, and overall physical performance^10^.

Exercise improves the BMD of the lumbar spine and femur in menopausal women, with consistent results. The most effective exercises use external loading and muscle tension to stimulate bone formation and maintain bone mass^11^. The only method that strengthens muscle, restores muscle characteristics, and reduces the risk of osteoporosis involves training the patient using various forms of exercise. Several exercise guidelines have been recommended to increase bone mass and muscle strength and reduce risk factors in PMW^12^. The best mode of exercise to perform and the time required to achieve the desired result are still unknown. For instance, the SIOMMS guidelines^13,14^ recommend 30 min per day of walking (in the open air, if possible) to reduce fall risk and indirectly affect vitamin D levels. The American College of Sports Medicine recommends that adults perform moderate-to-high intensity weight-bearing endurance activities such as tennis, stair climbing, and jogging, jumping activities such as volleyball and basketball, and resistance exercise such as weightlifting 3–5 times a week for 30–60 min, possibly in combination^6^.

Kinect-based VRT is a novel form of exercise that is gaining popularity for those with osteopenia. Kinect-based VRT creates an interactive virtual world from user motions. This fun, motivating exercise improves balance, coordination, and strength^15^. Virtual reality training (VRT) is frequently used to treat patients with neurosomatic balance dysfunction. It engages patients’ sensory feedback by presenting them with various virtual games. This training is better than other types of training since it is easier to use^16,17^.

Osteopenia or Osteoporosis is common among PMW in our society due to factors such as a sedentary lifestyle, lack of exercise, dietary habits, contraceptive use, and limited sunshine exposure due to traditional clothing in Pakistan. The rehabilitation professionals are exploring innovative and cost-effective methods, such as VRT, for managing PMW with Osteopenia due to limited time availability, expensive outpatient therapy, and increased use of costly electro-therapeutic equipment. Research is urgently needed to identify innovative treatments for PMW with Osteopenia to enhance BMD levels and delay progression to Osteoporosis. These methods can enhance adherence by motivating participants to exercise amusingly and engagingly. To the researcher's knowledge, no studies have been reported on the possible impacts of Kinect-based VRT in PMW with Osteopenia. Only one study^18^ has been conducted to investigate the effects of Kinect-based VRT on postmenopausal women with Osteoporosis, and it lasted for 12 weeks. Most studies on VRT in older women focused on assessing its impact on balance, fall risk, posture correction, and muscle strength in patients with neurological illnesses. Insufficient evidence exists on the effectiveness of Kinect-based VRT in PMW with osteopenia. There is a lack of specific literature focused on osteopenic PMW compared to those with osteoporosis. The hypothesis suggests that utilizing Kinect-based VRT significantly impacts BMD and fracture risk in PMW with osteopenia. Hence, the primary objective of this research was to assess the impact of 24 weeks of Kinect-based VRT on BMD and fracture risk in PMW with osteopenia.

## Results

**Figure 1:**
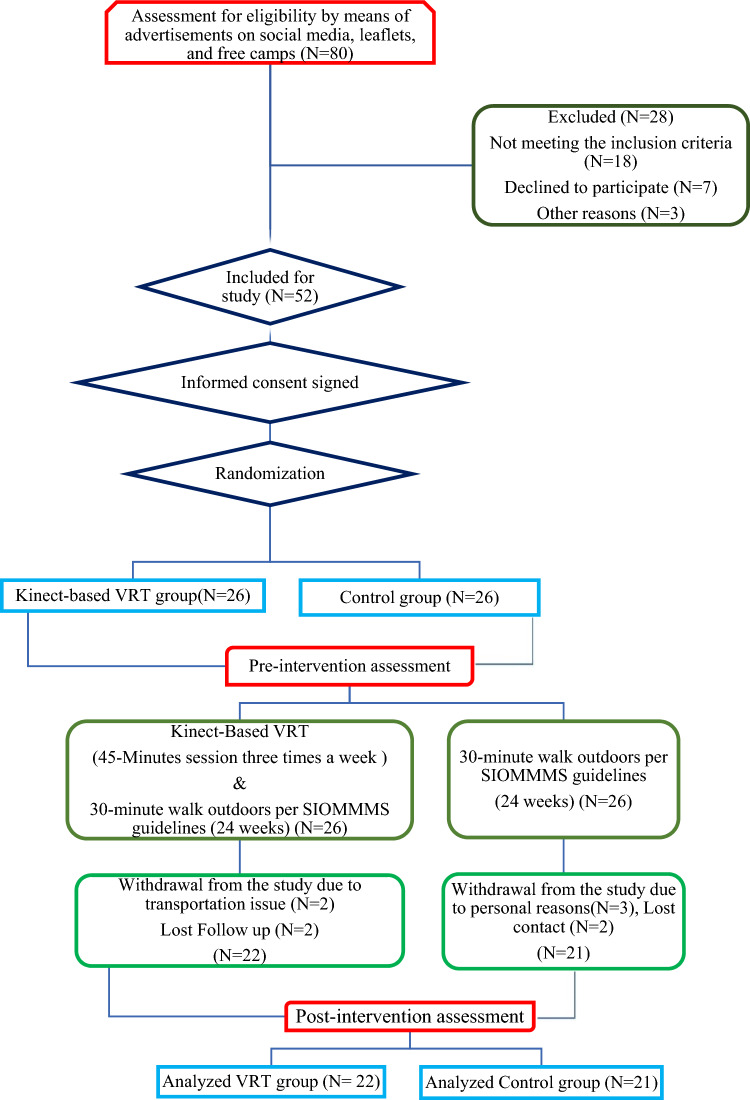
Consolidated standards of reporting trials (CONSORT) flow diagram.

After screening 80 PMW, 52 who met eligibility requirements were enrolled and randomized into two groups: Kinect-based VRT (n = 26) and control (n = 26). Table 1 lists the study participants' baseline characteristics. The baseline characteristics of the population analysed showed no significant differences between groups (*p* > 0.05). Figure 1 shows the CONSORT-compliant participant sample flow.

**Table 1:** Baseline characteristics of the participants.

Variables	Exp (n = 22) Mean ± SD	CON (n = 21) Mean ± SD	*p* value
Age (years)	58.3 ± 5.1	58.0 ± 5.5	0.9
Height (m)	1.64 ± 0.7	1.70 ± 0.02	0.06
Weight (kg)	73.2 ± 7.0	78.5 ± 6.0	0.63
BMI (kg/m^2^)	27.4 ± 2.5	27.5 ± 1.4	0.92
Age at Menarche (Years)	11.7 ± 0.9	11.5 ± 0.9	0.5
Age at Menopause (Years)	49.8 ± 1.6	49.6 ± 1.7	0.7
Waist Circumference (cm)	89.4 ± 4.1	92.1 ± 3.2	0.31
Hip Circumference (cm)	110.7 ± 4.5	114.0 ± 4.1	0.32
Waist-Hip Ratio	0.80 ± 0.1	0.81 ± 0.1	0.41
FES-1	23.3 ± 3.1	23.5 ± 2.2	0.76
Serum Calcium (mg/dl)	9.2 ± 0.6	9.0 ± 0.4	0.3
Serum 25-OH Vitamin D (ng/ml)	38.9 ± 6.0	38.8 ± 5.4	0.1
SCORE	8.5 ± 2.6	7.8 ± 1.6	0.3

Results are represented as the mean and standard deviation (±); *EXP* experimental group, *CON* control group, *SD* standard deviation, *BMI* body mass index; *FES-1* fall efficacy scale-1, *SCORE* simple calculated osteoporosis risk estimation.

Table 2 presents the representation of outcome variables that follow a normal distribution, wherein the mean value is accompanied by the standard deviation (SD) and the 95% confidence interval (CI). The BMD was substantially improved after 24 weeks at the Rt femoral neck (FN) in the experimental group. The difference was statistically significant (Cohen’s d = − 2.40, t = − 11.48, CI − 0.04, − 0.03, *p* =< 0.001), while in the control group, the difference was statistically significant (Cohen’s d = − 0.002, t = − 2.48, CI − 0.002, − 0.0001, *p* = < 0.022). The BMD was also improved after 24 weeks at the Lt FN in the experimental group. The difference was statistically significant (Cohen’s d = − 2.66, t = − 12.46, CI − 0.04, − 0.03, *p* = < 0.001), while in the control group, the difference was statistically significant (Cohen’s d = − 0.69, t = − 3.20, CI − 0.001, − 0.0003, *p* = < 0.004). BMD at the lumbar spine (LS) was improved significantly in the experimental group after 24 weeks. The difference was statistically significant (Cohen’s d = − 1.74, t = − 8.15, CI − 0.04, − 0.02, *p* = < 0.001); however, in the control group, BMD at the LS after 24 weeks was not significantly different (Cohen’s d = 0.13, t = 0.58, CI − 0007, − 0.01, *p* = 0.57). The FRAX (Rt HPMO) was reduced after 24 weeks in the experimental group with a statistically significant difference (Cohen’s d = 0.77, t = 3.60, CI 0.22, 0.83, *p* = 0.002), while in the control group, FRAX (Rt HPMO) was improved after 24 weeks and the difference was statistically significant (Cohen’s d = 0.62, t = 2.8, CI 0.007, 0.05, *p* = 0.01). The experimental group significantly improved the T score for both the right and left FN (*p* value < 0.001) compared to the control group (*p* value Rt FN = 104 and Lt FN = 0.162). The Z score (Rt FN) did not exhibit a statistically significant change, as shown by a *p* value > 0.05. However, the T score (*p* value < 0.05) at the lumbar spine was significantly changed in both groups.

**Table 2:** Within Group Comparison of BMD (Rt FN), T Score (Rt FN), Z Score (Rt FN), FRAX (Rt HPMO), BMD (Lt FN), T Score (Lt FN), BMD (LS) and T Score (LS).

Variable	Groups	N	Pretreatment Mean ± SD	Posttreatment Mean ± SD	ES Cohen’s d	95% CI		t*	*p* Value
						Lower	Upper		
BMD (Rt FN)	EXP	22	0.72 ± 0.052	0.76 ± 0.053	− 2.40	− 0.04	− 0.03	− 11.48	< 0.001
	CON	21	0.71 ± 0.04	0.72 ± 0.04	− 0.54	− 0.002	− 0.0001	− 2.48	0.022
T Score (Rt FN)	EXP	22	− 1.18 ± 0.41	− 0.86 ± 0.44	− 1.90	− 0.40	− 0.24	− 9.04	< 0.001
	CON	21	− 1.24 ± 0.33	− 1.2 ± 0.35	− 0.37	− 0.042	0.004	− 1.71	104
Z Score (Rt FN)	EXP	22	0.01 ± 0.75	0.06 ± 0.65	− 0.23	− 0.13	0.04	− 1.07	0.30
	CON	21	− 0.12 ± 0.83	− 0.13 ± 0.82	0.22	− 0.01	0.03	1.00	0.32
FRAX (Rt HPMO)	EXP	22	6.9 ± 1.7	6.4 ± 1.5	0.77	0.22	0.83	3.60	0.002
	CON	21	6.7 ± 1.4	6.7 ± 1.4	0.62	0.007	0.05	2.8	0.010
BMD (Lt FN)	EXP	22	0.71 ± 0.05	0.74 ± 0.05	− 2.66	− 0.04	− 0.03	− 12.46	< 0.001
	CON	21	0.71 ± 0.04	0.71 ± 0.04	− 0.69	− 0.001	− 0.0003	− 3.20	0.004
T Score (Lt FN)	EXP	22	− 1.30 ± 0.38	− 1.0 ± 0.40	− 2.03	− 0.37	− 0.23	− 9.51	< 0.001
	CON	21	− 1.20 ± 0.30	− 1.2 ± 0.30	− 0.32	− 0.02	− 0.004	− 1.45	0.162
BMD (LS.)	EXP	22	0.99 ± 0.12	1.02 ± 0.11	− 1.74	− 0.04	− 0.02	− 8.15	< 0.001
	CON	21	0.98 ± 0.17	0.99 ± 0.15	0.13	− 0.007	0.01	0.58	0.57
T Score (LS.)	EXP	22	− 0.98 ± 1.02	− 0.82 ± 0.95	− 1.08	− 0.22	− 0.09	− 5.08	< 0.001
	CON	21	− 1.09 ± 1.13	− 1.06 ± 1.13	− 0.62	− 0.05	− 0.008	− 2.82	0.01

Results are represented as the mean and standard deviation (±); *BMD* bone mineral density, *FN* femoral neck, *FRAX* fracture risk assessment tool, *HPMO* hip prediction of major osteoporotic, *LS* lumbar spine, *ES* effect size.

Table 3 presents a comparative analysis of the variables mentioned in Table 2, focusing on intergroup differences. The data used for this analysis are assumed to follow a normal distribution. No statistically significant difference was observed in the pretreatment values of the outcome variables between the two groups (*p* value > 0.05). This suggests that the variables in both groups were similar and exhibited homogeneity at baseline. After 24 weeks, BMD Rt FN (mean difference (MD) = 0.045, t = 3.16, CI 0.016, 0.074, *p* = 0.003), Lt FN (MD = 0.030, t = 2.25, CI 0.003, 0.054, *p* = 0.030) and LS (MD = 0.026, t = 0.62, CI − 0.058, 0.109, *p* = 0.539) exhibited that there was a significant difference between the groups in BMD (Rt & Lt femoral neck) and not a significant difference in BMD (LS) across both groups. There was no significant difference in FRAX (Rt HPMO) across both groups (MD = − 0.332, t = 0.75, CI − 1.221, 0.557, *p* = 0.455). T scores (Rt & Lt FN) exhibited significant differences between the two groups, with a *p* value < 0.05. However, there were no significant differences between the two groups observed in the Z score (Rt FN) and T score (LS), with a *p* value > 0.05. The two-factor analysis of variance (ANOVA) was conducted to analyse the effects of Group, Time, and their interaction on each dependent variable The group had significant main effects on BMD Rt FN (F = 8.26, *p* = 0.005, η^2^ = 0.09) and T Score Rt FN (F = 6.64, *p* = 0.012, η^2^ = 0.08), showing significant differences in mean scores. Significant main effects of Time were seen in BMD Rt FN (F = 2.94, *p* = 0.050, η^2^ = 0.04), T Score Rt FN (F = 4.06, *p* = 0.047, η^2^ = 0.05) and T Score Lt FN (F = 4.44, *p* = 0.038, η^2^ = 0.05). Group, Time, and interaction had no significant impacts on Z Score Rt FN, FRAX Score Rt HPMO, BMD Lt FN, BMD(LS) and T score (LS). No variable showed significant Group-Time interactions (*p* values 0.051–0.132). The results suggest that Group and Time independently influenced certain variables, but their effects did not interact.

**Table 3:** Group and time main effects and group*time interaction effects of BMD (Rt FN), T Score (Rt FN), Z Score (Rt FN), FRAX (Rt HPMO), BMD (Lt FN), T Score (Lt FN), BMD (LS) and T Score (LS).

Variable	Time	Experimental Group (n = 22)	Control Group (n = 21)	Mean difference (CI 95%)	Group main effects		Time main effects		Time*Group interaction effects	
					F	*P* value	F	*P* value	F	*P* value
BMD (Rt FN) (g/cm^2^)	Pretreatment	0.72 ± 0.052	0.71 ± 0.04	0.013 (− 0.02, 0.04)	8.26	0.005	2.94	0.050	2.56	0.113
	Posttreatment	0.76 ± 0.053	0.71 ± 0.04	0.045 (0.02, 0.07)						
T Score (Rt FN)	Pretreatment	− 1.18 ± 0.41	− 1.24 ± 0.33	0.066 (0.02, 0.29)	6.64	0.012	4.06	0.047	3.21	0.077
	Posttreatment	− 0.86 ± 0.44	− 1.2 ± 0.35	0.365 (0.12, 0.61)						
Z Score (Rt FN)	Pretreatment	0.01 ± 0.75	− 0.12 ± 0.83	0.137 (− 0.35, 0.63)	0.99	0.321	0.01	0.941	0.03	0.868
	Posttreatment	0.06 ± 0.65	− 0.13 ± 0.82	0.192 (− 0.27, 0.65)						
FRAX (Rt HPMO)	Pretreatment	6.9 ± 1.7	6.7 ± 1.4	0.167 (− 0.78, 1.11)	0.07	0.798	0.74	0.392	0.59	0.442
	Posttreatment	6.4 ± 1.5	6.7 ± 1.4	− 0.332 (− 1.22, 0.56)						
BMD (Lt FN) (g/cm^2^)	Pretreatment	0.71 ± 0.05	0.71 ± 0.04	− 0.0007 (− 0.03, 0.03)	2.31	0.132	2.94	0.090	2.56	0.114
	Posttreatment	0.74 ± 0.05	0.71 ± 0.04	0.030(0.003, 0.05)						
T Score (Lt FN)	Pretreatment	− 1.30 ± 0.38	− 1.20 ± 0.30	− 0.057 (− 0.26, 0.15)	1.43	0.235	4.44	0.038	3.90	0.051
	Posttreatment	− 1.0 ± 0.40	− 1.2 ± 0.30	0.233 (− 0.02, 0.45)						
BMD (LS) (g/cm^2^)	Pretreatment	0.99 ± 0.12	0.98 ± 0.17	− 0.005 (− 0.09, 0.08)	0.11	0.740	0.18	0.68	0.27	0.61
	Posttreatment	1.02 ± 0.11	0.99 ± 0.15	0.026 (− 0.06, 0.11)						
T Score (LS)	Pretreatment	− 0.98 ± 1.02	− 1.09 ± 1.13	0.104 (− 0.563, 0.770)	0.55	0.46	0.17	0.68	0.08	0.78
	Posttreatment	− 0.82 ± 0.95	− 1.06 ± 1.12	0.234 (− 0.41, 0.88)						

Results are represented as the mean and standard deviation (±); *BMD* bone mineral density, *FN* femoral neck, *FRAX* fracture risk assessment tool, *HPMO* hip prediction of major osteoporotic, *LS* lumbar spine.

Table 4 shows the within-group comparison of outcome variables with data that were not normally distributed (*p* value < 0.05). The FRAX Rt HFP score improved after 24 weeks in the experimental group; the difference was statistically significant (Cohen’s d = 0.85, Z = − 3.30, *p* = < 0.001), and the control group after 24 weeks was also significantly different (Cohen’s d = 0.47, Z = − 2.0, *p* = 0.05). FRAX Lt HFP score improved after 24 weeks in the experimental group; the difference was statistically significant (Cohen’s d = 1.40, Z = − 4.0, *p* =  < 0.001), while that in the control group after 24 weeks was not significantly different (Cohen’s d = 0.19, Z = − 0.45, *p* = 0.66). The FRAX Lt HPMO score improved after 24 weeks in the experimental group; the difference was statistically significant (Cohen’s d = 1.01, Z = − 4.12, *p* =  < 0.001), while that in the control group after 24 weeks was not significantly different (Cohen’s d = , Z = − 1.13, *p* = 0.26). The Z scores (Lt FN and LS) showed no significant change in either group, with a *p* value of > 0.05.

**Table 4:** Within-group Comparison of FRAX (Rt HFP), Z score (Lt FN), FRAX(Lt HFP), FRAX (Lt HPMO), and Z score(LS).

Variable	Groups	N	Pretreatment Mean ± SD	Posttreatment Mean ± SD	Median		ES Cohen’s d (95% CI)	Z value	*p* value
					Pretreatment	Posttreatment			
FRAX (Rt HFP.)	EXP	22	0.50 ± 0.31	0.37 ± 0.25	0.35	0.20	0.85(0.35,1.33)	− 3.30	< 0.001
	CON	21	0.51 ± 0.26	0.49 ± 0.26	0.50	0.40	0.47(0.02,0.92)	− 2.0	0.05
Z Score (Lt FN)	EXP	22	− 0.27 ± 0.72	− 0.05 ± 0.61	0.20	0.10	0.18 (− 0.24, − 0.6)	− 0.73	0.47
	CON	21	− 0.18 ± 0.88	− 0.17 ± 0.88	0.10	0.10	− 0.15 (0.58,0.28)	− 0.71	0.48
FRAX (Lt HFP)	EXP	22	0.58 ± 0.32	0.39 ± 0.23	0.40	0.30	1.40 (0.80,1.99)	− 4.00	< 0.001
	CON	21	0.51 ± 0.25	0.51 ± 0.24	0.50	0.50	0.19 (− 0.24,0.63)	− 0.45	0.66
FRAX (Lt HPMO)	EXPMO	22	7.15 ± 1.62	6.52 ± 1.51	6.95	6.55	1.01 (0.49,1.52)	− 4.12	< 0.001
	CON	21	6.38 ± 2.13	6.36 ± 2.10	7.00	7.00	0.25 (− 1.89,0.68)	− 1.13	0.26
Z Score (LS)	EXP	22	0.46 ± 1.18	0.45 ± 1.10	0.55	0.3	0.08 (− 0.33,0.49)	− 0.38	0.71
	CON	21	0.77 ± 2.10	0.76 ± 2.10	0.90	0.80	0.178 (0.26,0.61)	− 0.82	0.41

Results are represented as the mean and standard deviation (±); *FRAX* fracture risk assessment tool, *HFP* hip fracture prediction, *HPMO* hip prediction of major osteoporosis, *ES* effect size.

Table 5 shows the between-group comparison of the variables mentioned in Table 4. No statistically significant difference was observed in the pretreatment values of the outcome variables between the two groups (*p* value > 0.05). This suggests that the variables in both groups were similar and exhibited homogeneity at baseline. After 24 weeks, FRAX Rt HFP [Mean Difference (MD) = 0.43 ± 0.26, Mean Rank (exp = 18.27, con = 25.90), Med = 0.30, Z = − 2.02, *p* = 0.04] showed a significant difference across the groups, while FRAX Lt HFP [MD = 0.44 ± 0.24, MR (exp = 18.57, con = 25.60), Med = 0.40, Z = − 1.86, *p* = 0.06] and FRAX Lt HPMO [MD = 6.44 ± 1.80, MR (exp = 21.80, con = 22.21), Med = 6.6, Z = − 0.11, *p* = 0.91] showed no significant difference across the groups. The Z scores of Lt FN and LS were also not significantly different across the groups, with *p* = 0.47 and 0.41, respectively. Significant main effects of Time were seen in FRAX Score Lt HFP (F = 2.96, *p* = 0.050, η^2^ = 0.04). Group, Time, and interaction had no significant impacts on FRAX Score Rt HFP, Z Score Lt FN, FRAX Score Lt HPMO and Z score(LS). No variable showed significant Group-Time interactions (*p* values 0.051–0.132). The results suggest that Group and Time independently influenced certain variables, but their effects did not interact.

**Table 5:** Group and time main effects and group*time interaction effects of FRAX (Rt HFP), Z score (Lt FN), FRAX(Lt HFP), FRAX (Lt HPMO), and Z score(LS).

Variable	Time	Mean + SD (n = 43)	Mean ranks		Median (n = 43)	Group main effects		Time main effects		Group*Time interaction effects	
			EXP (n = 22)	CON (n = 21)		F	*P* value	F	*P* value	F	*P* value
FRAX (Rt HFP)	Pretreatment	0.51 ± 0.28	20.93	23.12	0.40	1.12	0.293	1.68	0.198	0.95	0.333
	Posttreatment	0.43 ± 0.26	18.27	25.90	0.30						
Z Score (Lt FN)	Pretreatment	− 0.10 ± 0.80	23.30	20.64	0.10	0.65	0.42	0.003	0.96	0.01	0.91
	Posttreatment	− 0.11 ± 0.75	22.75	21.21	0.10						
FRAX (Lt HFP)	Pretreatment	0.55 ± 0.29	22.82	21.14	0.50	0.21	0.65	2.96	0.05	2.70	0.10
	Posttreatment	0.44 ± 0.24	18.57	25.60	0.40						
FRAX (Lt HPMO)	Pretreatment	6.77 ± 1.90	23.98	19.93	7.0	1.32	0.25	0.65	0.42	0.59	0.12
	Posttreatment	6.44 ± 1.80	21.80	22.21	6.6						
Z Score (LS)	Pretreatment	0.62 ± 1.70	21.07	22.98	0.90	0.71	0.40	0.001	0.98	0.002	0.99
	Posttreatment	1.49 + 0.51	21.16	22.88	0.70						

Results are represented as the mean and standard deviation (±); *FRAX* fracture risk assessment tool, *HFP* hip fracture prediction, *HPMO* hip prediction of major osteoporosis.

**Figure 2:**
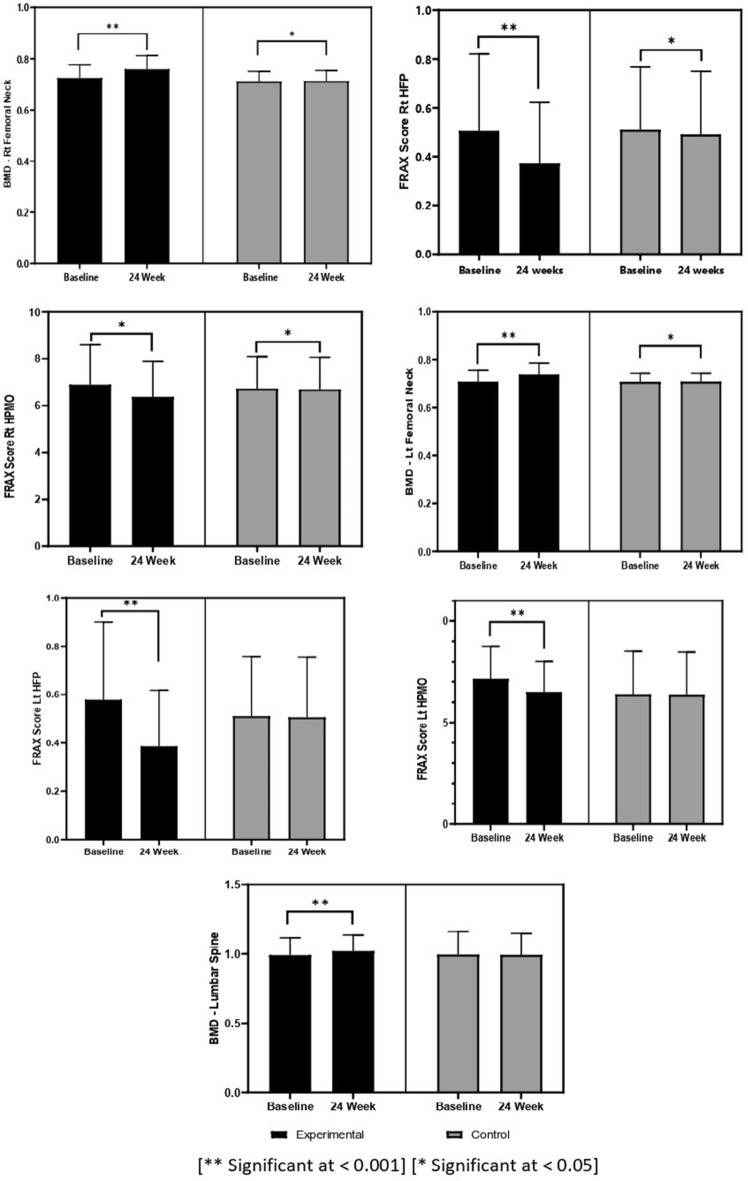
The differences between mean BMD and FRAX score (HFP & HPMO) values in the experimental and control groups, Abbreviations: *BMD* bone mineral density, *FRAX* fracture risk assessment tool, *HFP* hip fracture prediction, *HPMO* hip prediction of major osteoporotic.

Figure 2 shows that BMD at both the Rt and Lt femoral neck was significantly improved from baseline to after 24 weeks with a *p* value < 0.001 compared to the control group with a *p* value < 0.05. BMD at the lumbar spine was substantially increased in the experimental group, with a *p* value < 0.001, compared to the control group, with a *p* value > 0.05. FRAX HFP (Rt & LT FN) was significantly improved with a *p* value < 0.001 compared to the control group, with a *p* value < 0.05 for Rt FN and > 0.05 for Lt FN. The FRAX HPMO scores for Rt and Lt FN significantly improved, with *p* values of < 0.001 and < 0.05, respectively, compared to the control group, with *p* values of < 0.05 for Rt FN and > 0.05 for Lt FN.

**Figure 3:**
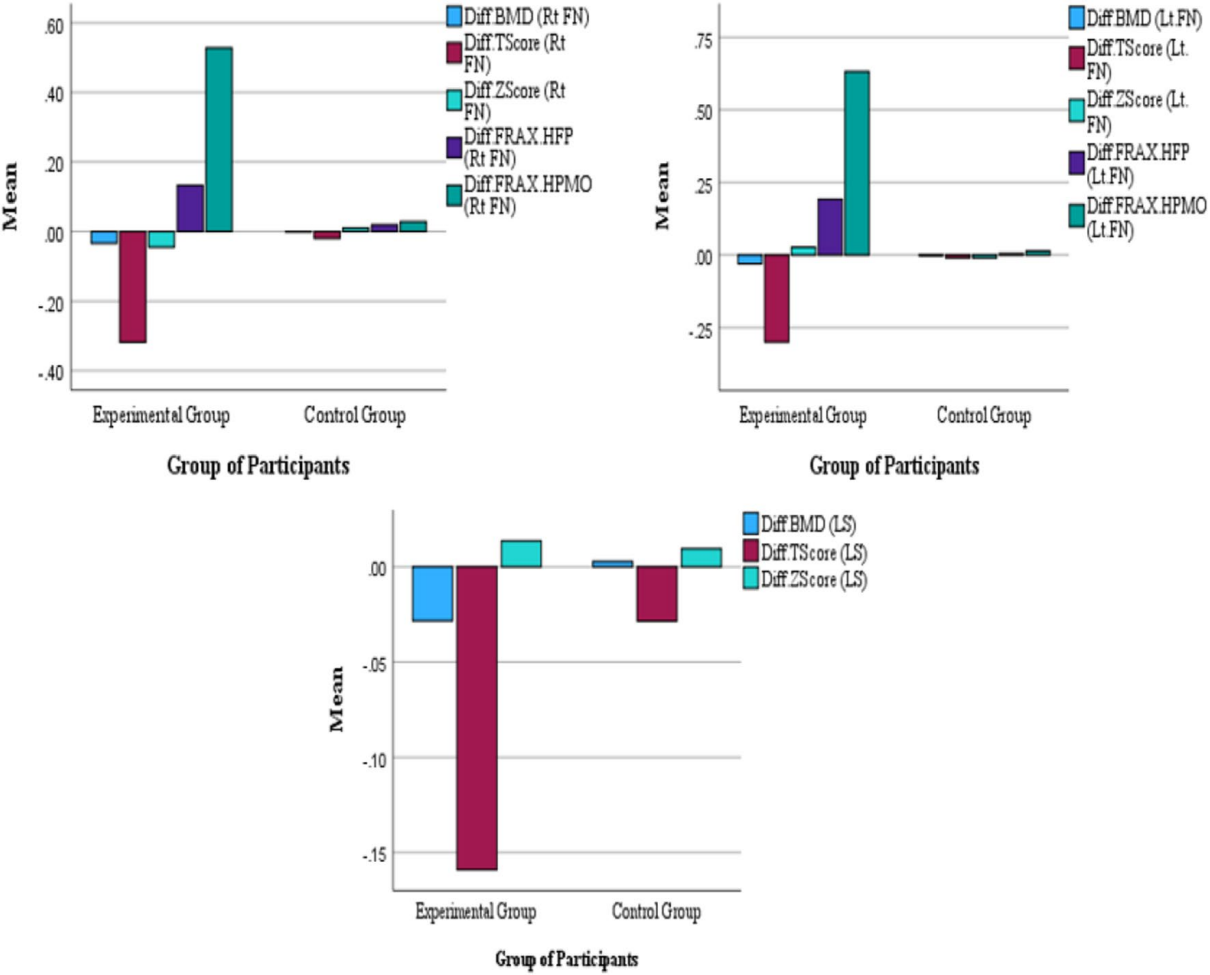
Group-wise Minimal Clinically Important Differences (MCID) between BMD and FRAX score (HFP & HPMO) at FN (Rt & Lt) and Lumbar spine, Abbreviations: *BMD* bone mineral density, *FRAX* fracture risk assessment tool, *HFP* hip fracture prediction, *HPMO* hip prediction of major osteoporotic.

Figure 3 illustrates the group-wise Minimal Clinically Important Differences (MCID) between BMD and FRAX score (HFP & HPMO) at FN (Rt & Lt) and Lumbar spine.

## Discussion

The present study examined the effects of Kinect-based VRT on BMD and fracture risk in postmenopausal women with osteopenia. The current era has witnessed the emergence of technological developments, specifically in the realm of virtualization of rehabilitation. This technology plays a significant role in evaluating, managing, and investigating patients^19^. PMW faces increased susceptibility to osteopenia and osteoporosis because of the gradual decrease in oestrogen levels. Physical activity plays a crucial role in preserving bone health, and VRT presents an enjoyable and captivating exercise modality. The present study's results demonstrate notable impacts of Kinect-based VRT on the BMD of the right and left femoral necks and the lumbar spine.

Additionally, fracture risk was considerably reduced at both femoral necks. Regardless of calcium and vitamin D intake, following 24 weeks of intervention, the participants’ BMD levels were maintained or even increased. The findings of García-Gomariz et al. ^20^ study support the notion that regular physical exercise may positively impact maintaining or enhancing BMD in PMW with osteopenia or osteoporosis. These effects appear to be independent of vitamin D and calcium supplementation^20^.

Nambi et al.^21^ examined how Kinect-based VRT affected BMD and BMC in osteoporosis-afflicted PMW. Participants played a shooting game on an Xbox 360 with a Kinect sensor. The participant could perform hip and knee movements within her physical restrictions. Progressive exercise increases muscle activation and challenge. The 12-week training program had a daily duration of 45 min and a weekly frequency of four days. The study found that VRT improved BMD, bone mineral content, and quality of life in PMW with osteoporosis. Furthermore, this training modality may decrease the likelihood of experiencing osteoporotic fractures^21^. In the present study, Kinect-based VRT was employed to examine its effects on BMD and fracture risk in a population of PMW diagnosed with osteopenia. The VRT protocol was executed sequentially, comprising three distinct stages: warm-up, activity phase, and cool-down. Each stage was supported by an evidence-based selection of X-Box 360 Kinect games. The training regimen needed 45 min per day and was conducted thrice weekly for 24 weeks, and this intervention duration in our trial was longer than in prior similar study.Both experimental and control groups were also engaged in a 30-min daily outdoor walk per SIOMMMS. Our research findings indicate that VRT yields extensive BMD and fracture risk advantages. However, it is essential to note that the current study’s population consisted exclusively of osteopenic PMW and a treatment duration of 24 weeks showed better results in BMD and fracture risk.

Concerning the optimal kind of physical activity for improving BMD, numerous research studies have shown that exercise therapies incorporating a combination of high-impact exercises, flexibility exercises, and resistance training yield superior outcomes in terms of sustaining regular physical activity compared to conventional or placebo treatments^22,23^. The current study also observed similar findings, as the Kinect-based VRT protocol incorporates games from Your Shape Fitness Evolved, Kinect Sports, and Kinect Adventures. These games have been shown to provide various benefits to PMW with osteopenia, and they include a combination of high-impact, low-impact, flexibility, strength, and balance exercises.

The International Osteoporosis Foundation and other reputable organizations recommend incorporating weight-bearing activities/exercises as a preventive measure against osteopenia and osteoporosis. Various forms of weight-bearing exercises exist, encompassing high-impact activities such as running and jumping and low-impact activities such as body-weight training, water aerobics, and walking^24^. Recent reports indicate a considerable improvement in trochanter and femoral neck BMD following high-impact weight-bearing exercise among premenopausal women^25^. Furthermore, the implementation of high-impact weight-bearing and resistance training, whether in conjunction or independently, increased BMD by approximately 1–2% at the femoral neck and lumbar spine in both premenopausal women and PMW. However, the influence on BMD at other anatomical sites was minor^26^. Previous research has yielded comparable findings, indicating that engaging in high-impact exercise three times per week over 12 months led to a noteworthy enhancement in femoral neck BMD ranging from 0.6 to 1.1% among premenopausal women^27,28^. The present study's results are consistent with the mentioned studies, which indicate that the effects of Kinect-based VRT exercises are comparable to other weight-bearing and high-impact exercises, such as running, jumping, hiking, and skiing. The participants in this study demonstrated an improvement in bone BMD after a 24-week intervention period. Specifically, BMD increased from 0.72 to 0.76 at the right femoral neck, 0.71–0.74 at the left femoral neck, and 0.99–1.02 at the lumbar spine. However, it should be noted that the observed increase in BMD in this study did not reach the levels reported in the above studies.

Previous studies have used exercise utilizing VR technology in community and home-based settings. The findings indicated that the use of VR as an exercise modality is another possible option for physical training, and it significantly improved the static and dynamic equilibrium in senior citizens. Additionally, senior citizens were consistently stimulated by VR fitness game workouts, which consistently motivated them to engage in various exercise types. This intensifies training and improves balance, which is critical for preventing falls and fractures in older adults^29–31^. The present study incorporated Kinect-based VRT in a sample of PMW patients with osteopenia. Including entertaining and motivating games within the study protocol positively influenced the participants’ adherence to the study protocol. Following a 24-week interventional period, a notable reduction in the FRAX score, namely, in the domains of HFP and HPMO, was observed.

Multicomponent training combines aerobics, strengthening, progressive resistance, balancing, and dance to increase or maintain bone mass. Different exercise types can significantly improve BMD at the femoral neck, greater trochanter, and spine level^32^. Nikander et al. meta-analysis in postmenopausal women showed positive findings on the effects of exercise. Resistance training positively affects lumbar bone mineral density (BMD), but when combined with low to moderate-impact workouts such as jogging, walking, and stair climbing, it is more effective in preserving BMD in both the lumbar and femoral regions^33^. The present study combined Kinect-based Virtual Reality Training, a blend of multiple games, with a 30-min daily outdoor walk per SIOMMMS guidelines. This combination resulted in a notable improvement in BMD in the lumbar spine and femoral neck and decreased fracture risk.

The observed progress of the Kinect-based VRT group after 24 weeks aligns with the research conducted by Abeer M. ElDeeb. ElDeeb’s study demonstrated that a 24-week whole-body vibration (WBV) training program enhanced leg muscle function and increased BMD in the lumbar and femoral regions of postmenopausal women with low BMD—consequently, the likelihood of experiencing falls and consequent fractures diminished^34^.

The strengths of the study were as follows: (a) the utilization of an innovative and potentially captivating intervention that has the potential to enhance adherence to an interventional program; (b) the approach exhibited promising potential in improving bone health and mitigating the likelihood of fractures among a group that was particularly susceptible to osteoporosis-related issues; and (c) the evaluation of the intervention was enhanced by using objective measures of bone health and ten-year probability of fracture risk, such as BMD and fracture risk assessments (FRAX).

Nonetheless, the study had several limitations. First, confounding factors, such as medication use, food, and comorbidities, could impact bone health and the risk of fractures. The feasibility and effectiveness of the intervention could have been impacted by any technological limits or challenges associated with the Kinect-based VRT. It is advised that the following studies should be conducted with a long-term follow-up period to assess the sustainability of the outcomes: a comparative analysis of the Kinect-based VRT protocol and alternative treatment modalities/protocols in PMW with osteopenia and a comparative study between PMW with osteopenia and osteoporosis.

## Conclusion

The research findings indicate that a Kinect-based VRT intervention resulted in significantly improved BMD and a reduced fracture risk, as predicted by HFP and HPMO measurements. These improvements were more pronounced in the experimental group than in the control group. Thus, Kinect-based VRT may be utilized as an effective intervention to improve BMD and reduce fracture risk in postmenopausal women with osteopenia.

## Methods

### Study design and setting

This prospective, randomized, double-blinded controlled trial with parallel groups was conducted in 2021–2023 at Riphah Rehabilitation Center, Riphah International University, Lahore, Pakistan and Genesis Healthcare Consultants, Lahore, Pakistan. The clinical trial protocol was ethically approved by The Research and Ethics Committee, Riphah College of Rehabilitation and Allied Health Sciences, Riphah International University, Lahore, Pakistan (REC/RCR&AHS/21/1102) and was carried out in compliance with the Helsinki Declaration. The clinical trial (NCT04862910) was registered in the Clinical Trials.gov Protocols Registration and Results System on 28/04/2021. The study followed the CONSORT 2010 Statement (Consolidated Standards of Reporting Trials)^35^.

### Sample size calculation

Nonprobability convenience sampling was used to recruit study participants. Pilot study data determined the sample size. The pilot study included twenty participants with mean lumbar spine BMD in the experimental (0.03 ± 0.02) and control (0.007 ± 0.032) groups. The pilot study yielded a 0.9 effect size. A sample size of 42 was determined using an effect size of 0.9, 80% power, 95% confidence interval (CI), and 5% margin of error. Following the inclusion of a 20% attrition rate, a sample size of 52 was determined, and 26 participants were allocated to each group.

### Study participants

PMW were screened at free bone health camps via posters, leaflets, social media campaigns, and outpatient physical therapy clinics. Females with any of the aforementioned risk factors as per the International Osteoporosis Foundation (IOF) were chosen for the initial screening^36^. being 45 years of age or older; having menopause or a hysterectomy before that age; having early postmenopausal (1–8 yr) status; having the ovaries removed before menopause, oestrogen deficiency or amenorrhea; height loss; history of fractures or a family history of low BMD; low BMD and DEXA scan recommended by Simple Calculated Osteoporosis Risk Estimation (SCORE) score > 6^37^; normal serum calcium level of 8.6–10.3 mg/dL; and a minimum level of 30 ng/mL of serum 25-OH vitamin D value^38^. This study included PMW with the following characteristics: between the ages of 48 and 70 years; osteopenia confirmed by dual-energy X-ray absorptiometry; lumbar spine or femur T score between − 1 and − 2.5; recommended by the study investigators or confirmed by a doctor within the previous 12 months; current body mass index (BMI) 30 kg/m^2^; patients with normal balance and no risk of falling assessed by the Tandem Stance Test (TST); Fall Efficacy scale (FES-I) value between 18 and 28 to rule out fear of fall^39^; and those with or without pharmaceutical treatment of osteopenia. Patients with severe impairment of sensory and/or communicative abilities, impaired cognition, unstable angina, lung conditions needing oxygen therapy, any serious medical condition, neurologic problems, history of virtual game therapy within the last six months, virtual game phobia, symptomatic orthostatic hypotension (diastolic > 95 mmHg, systolic > 160 mmHg), secondary osteoporosis, arthrosis, osteoporotic fractures that are known to have occurred, neoplastic illness, and epilepsy were excluded. An impartial assessor assessed all individuals enrolled in the initial assessment to determine if they met the criteria for inclusion in the study. The participants were evaluated twice during the study, once at the beginning and again six months after the intervention. Before conducting the baseline evaluation, participants were instructed to familiarize themselves with the study procedure and provide informed consent by signing a consent form. The participants were informed that they could withdraw from the research without facing any negative consequences and were actively encouraged to seek clarification by posing inquiries.

### Randomization and blinding

Participants who signed the informed consent form and agreed to participate in the study were randomly assigned to one of two groups using a computer-generated randomization table. The sequentially numbered, opaque, sealed envelopes (SNOSE) approach was utilized for allocation concealment. An independent researcher with no clinical involvement devised the envelopes. Except for the therapist administering treatment, all other personnel, including the assessor and participants, were blinded to the treatment.

### Groups and intervention procedures

Participants were assigned to either Group A (Experimental) or Group B (CON). Group A (Experimental) participated in the Kinect-based VRT for 45 min a day, three times per week on alternate days for 24 weeks. Additionally, they were instructed to engage in a daily 30-min walk outdoors, following the guidelines provided by the Societa’ Italiana dell'Osteoporosi, del Metabolismo Minerale e delle Malattie dello Scheletro (SIOMMMS), which is the Italian Society for Osteoporosis, Mineral Metabolism, and Bone Diseases^13,14^. Group B (CON) also received instructions to engage in a daily 30-min walk outdoors, following the SIOMMMS guidelines. However, they were not provided with systematic and supervised exercises during the same time frame and were instead instructed to maintain their regular active lifestyle^40,41^. The participants’ monthly visits and phone calls were adjusted to maintain regular communication^42^.

### Kinect-based VRT protocol

Xbox Kinect (Xbox 360 S CONSOLE, Microsoft Corporation M-1439, Redmond, WA, USA) was utilized for Kinect-based VRT. The Xbox Kinect platform does not require the user to carry an interface device and instead employs an infrared camera to capture users’ body movement in 3D space for involvement in in-game activities. Instead, the user's body acts as the game controller in 3D space. The screen and Kinect sensor were set up in a room free of external influences for the training. The sensor was placed, and the participants took part while standing 1.5–2 m from the screen^43^.

**Figure 4:**
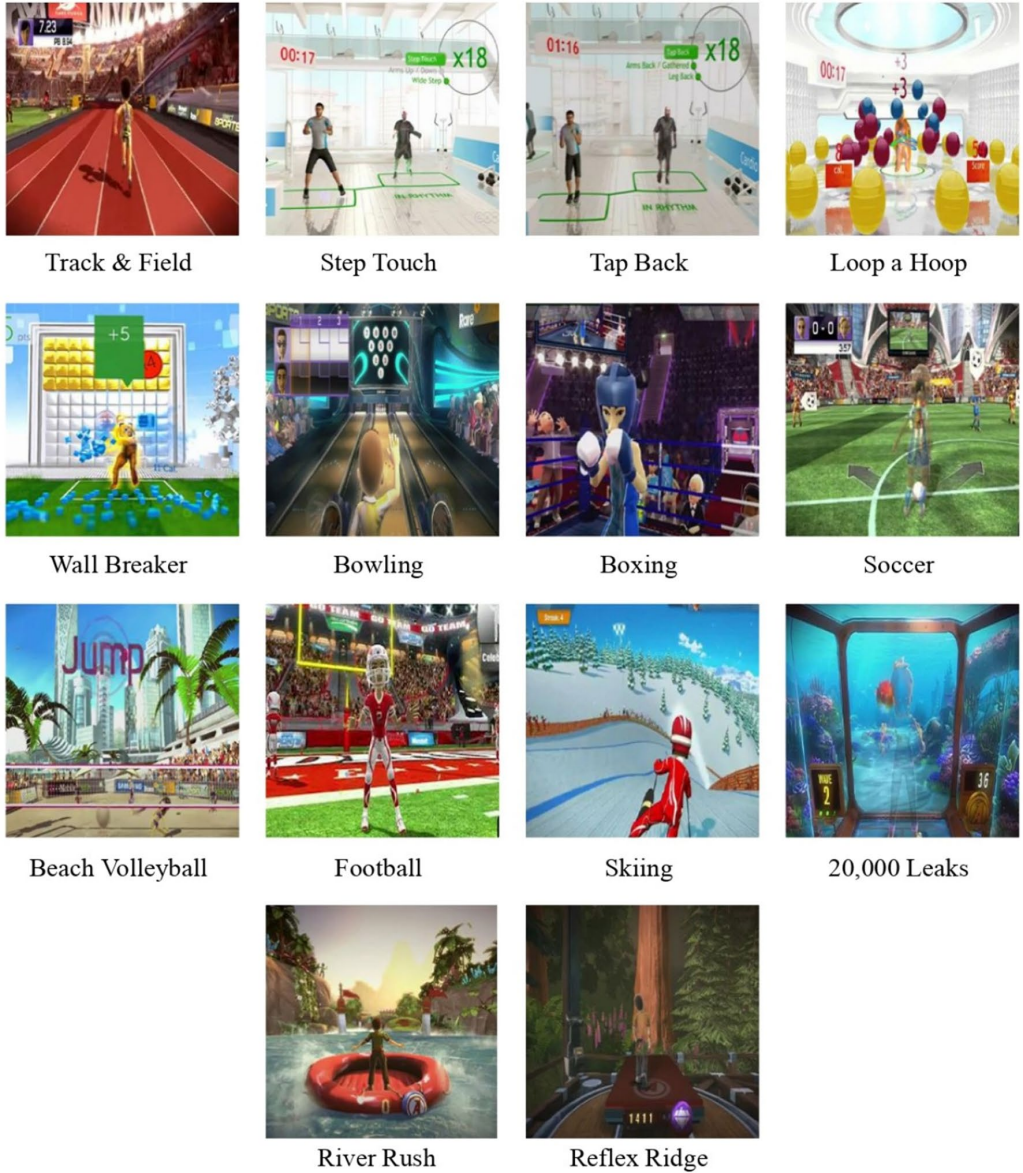
Games used in kinect-based VRT protocol.

**Figure 5:**
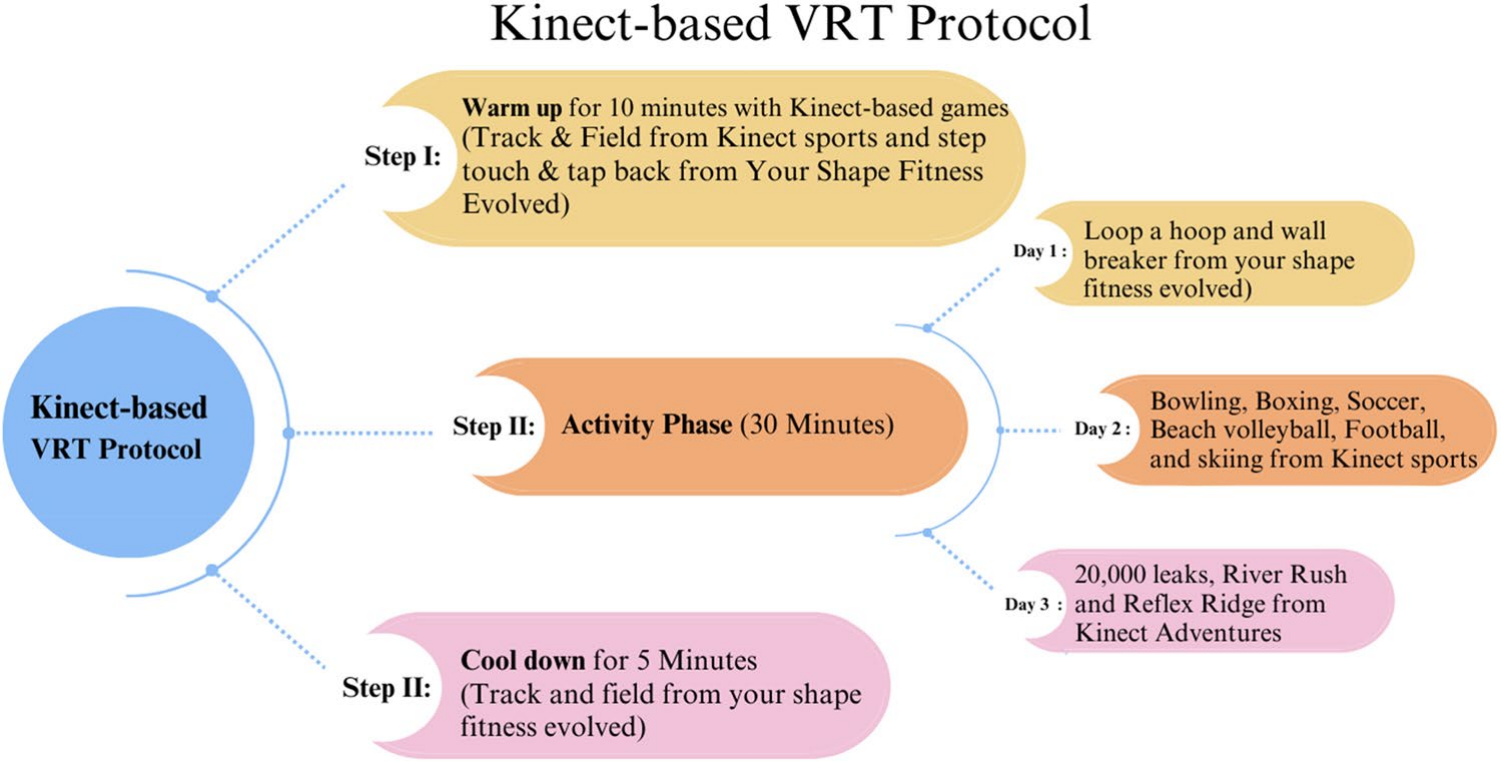
Kinect-based VRT protocol.

Based on prior literature, games were selected for three days a week. Upper and lower body strength and high-impact training were considered when choosing the Xbox Kinect 360 game programs, which included games from Your Shape Fitness Evolved, Kinect Sports, and Kinect Adventures, which increasingly involved the muscles of the lower extremities, upper limbs, and torso as the intensity of the work in the game was increased^43–48^ (Fig. 4). The participants played each game for the allotted amount of time throughout the 45 min of Xbox Kinect video game training on alternate days for 3 days/week for 24 weeks, under the guidance of the therapist monitoring the performance. The Kinect-based VRT protocol was clinically implemented in three steps **(**Fig. 5).

The use of different exercises on different days of the week delighted and motivated the participants because repeating the same exercises might have bored them. As the participants improved, the exercises became more difficult. The LED screen displayed an animated character that moved like the player did during a game. Player movement was controlled by viewing characters to score high. Participants were told their score from the previous game and encouraged to improve upon it in the next session. Before game training and when asked, the therapist let participants choose a game while monitoring their safety.

### Outcome measures

#### Anthropometric measurements

Body mass index (BMI), which is determined by dividing body weight (kg) by the square of body height (m^2^), was measured along with body height and weight. A stadiometer (TKK 11,253, Takei Scientific Ins Co., Tokyo, Japan) was used to measure body height to the nearest 0.1 cm. A balancing beam scale (Seca 700, Seca Co., Hamburg, Germany) was used to measure body weight to the nearest 0.1 kg.

#### Dual-energy X-ray absorptiometry (DEXA)

The most common gold standard test for BMD, T score, and Z score is DEXA (dual-energy X-ray absorptiometry), which can be evaluated in vivo and has been validated by several studies for fracture risk assessment^49^. DEXA measurements are most frequently made at the proximal femur (including the femoral neck) and lumbar spine (L1–L4) to determine the risk of osteoporosis^50^. This study utilized a dual-energy X-ray absorptiometry (DEXA) scan (PRIMUS) to obtain baseline measurements of the BMD in the femoral neck and lumbar spine, expressed in grams per square centimetre (g/cm^2^). The error coefficients for the lumbar spine and femoral neck were between 1 and 2.5%^51^. The coefficient of variation for the lumbar vertebrae was 1.12%, and for the proximal femur, it was 1.86% ^52,53^.

#### WHO's fracture risk assessment tool (FRAX) algorithm

The FRAX tool was developed to assess patients' fracture risk. It is based on individual patient models incorporating the hazards associated with clinical risk variables and femoral neck BMD. It computes the 10-year probability of osteoporotic fracture using the WHO’s fracture assessment calculator, age, sex, weight, height, clinical risk factors, and femoral neck BMD results^54^.

### Statistical analysis

Data entry and statistical analysis were performed in IBM SPSS 25. Analyses employed mean, median, variance, and standard deviation. The Shapiro‒Wilk test determined data normality. Parametric tests (paired-samples T tests for within-group comparison, independent-samples T tests for Between-group comparison) were used for variables (BMD Rt FN, T score Rt FN, Z score Rt FN, FRAX Rt HPMO, BMD Lt FN, T score Lt FN, BMD LS and T score LS) with a normal distribution and significance value > 0.05, while nonparametric tests (Wilcoxon signed rank test for within-group comparison, Mann‒Whitney test for Between-group comparison) were used for variables(FRAX Rt HFP, Z score Lt FN, FRAX Lt HFP, FRAX Lt HPMO and Z score LS) with nonnormal distributions with < 0.05 significance value. Mean score changes determined the successful intervention. Data significance was < 0.05. The two-way analysis of variance (ANOVA) was conducted to analyze the effects of Group (experimental and Control), Time (Baseline,24 weeks), and their interaction on each dependent variable. Due to 4 experimental and 5 control group dropouts, 22 experimental and 21 control-group subjects were evaluated.

## Ethical approval

The clinical trial protocol was ethically approved by The Research and Ethics Committee, Riphah College of Rehabilitation and Allied Health Sciences, Riphah International University, Lahore, Pakistan (REC/RCR&AHS/21/1102) and was carried out in compliance with the Helsinki Declaration. The clinical trial (NCT04862910) was registered in the Clinical Trials.gov Protocols Registration and Results System.

## Data Availability

The data generated or analysed throughout this study are outlined in this paper and can be made available by the corresponding author upon reasonable request.
